# Editorial: Array-Based Sensing Techniques for Clinical, Agricultural Biotechnology, and Environmental Analysis

**DOI:** 10.3389/fchem.2021.654707

**Published:** 2021-03-12

**Authors:** Zheng Li, Qingshan Wei, Jinsong Han

**Affiliations:** ^1^ Institute for Advanced Study, Shenzhen University, Shenzhen, China; ^2^ Department of Chemical and Biomolecular Engineering, North Carolina State University, Raleigh, NC, United States; ^3^ State Key Laboratory of Natural Medicines/National R&D Center for Chinese Herbal Medicine Processing, College of Engineering, China Pharmaceutical University, Nanjing, China

**Keywords:** array-based sensing, pattern-recognition, optoelectronic nose, optical sensing, biosensing

The array-based sensors, as a class of easy-to-use analytical tools that tracks the pattern-based responses, has emerged as a versatile method for the identification and quantification of chemically diverse analytes, and demonstrated their usefulness in addressing a wide range of analytical challenges (Albert et al., 2000; Han et al., 2017; Li et al., 2019). By using chemically diverse chemoresponsive dyes or biological receptors in combination with chemometric analysis, sensor arrays can be constructed to discriminate various structurally similar analytes or their mixtures. While many biological systems employ a highly specific lock-and-key strategy for molecular recognition, the array-based approach provides an alternative means of creating specificity through pattern recognition of the response from an assembly of cross-reactive sensors, which can be applied to both gaseous and aqueous analytes (Sun et al., 2020). The primary feature of an advanced sensor array, as an analog to the mammalian nose, is that it gives a composite response to target mixtures and provides accurate discrimination between highly similar complex mixtures without the need to know individual components (Potyrailo et al., 2015).

To present state-of-the-art research in this field, we launched a Research Topic in Frontiers in Chemistry entitled “Array-Based Sensing Techniques for Clinical, Agricultural Biotechnology, and Environmental Analysis”. This Research Topic included 5 articles, including 1 original research article and 4 reviews, which covered the use of colorimetric, fluorometric, and electrochemical sensor arrays for bioengineering monitoring, bioanalysis, and many other clinical applications. The original research article from Mohs et al. reported a ratiometric sensor array that enabled the prediction and quantification of complex bacterial mixtures; the method was able to quantify individual components with the accuracy of ∼80% and without the need for acquisition of new reference data or expansion of the training datasets. This approach significantly enlarged the functionality of optical sensor arrays and provided essential insights into data processing for the analysis of biologically complex samples. The other 4 review articles summarized nearly all aspects of the sensor array applications in fundamental research or industrial production. Chen et al. introduced the recent progress of employing aggregation-induced emission luminogens (AIEgens) as fluorescent sensors and in building up sensor arrays for the identification of biological analytes, including biomolecules and bacteria. Examples were incorporated to illustrate the probe design and working mechanism, capability of the sensor array, as well as the clinical implications of these multidimensional fluorescent sensing methods. Li et al. reviewed the state-of-the-art analytical technology for bioprocess monitoring of volatile molecular markers, and particularly described the advantages of electronic or optoelectronic noses over single-element chemical sensors that could target a more diverse range of key nutritious components, products or potential contaminants produced from various biomanufacturing processes. The key to the improvement of sensing performance is the feature that the sensor array aims to probe primarily the chemical reactivity of analytes, rather than physical properties. This principle provided a high dimensionality for biosensing purposes and enabled high sensitivity and remarkable discrimination capability even among highly similar targets in gaseous phases. Ding et al. focused on the construction of different optical sensor arrays for protein discrimination and their applications in real clinical samples. Three strategies for building such sensor arrays were introduced, including multi-element-based sensor arrays, environment-sensitive sensor arrays and multi-wavelength-based single sensing systems. The authors attempted to explain the connections and distinctions among different strategies, and particularly emphasized that the third type is more attractive due to the ease of data collection and less consumption of samples. Li et al. summarized the recent advances in the fabrication of micro/nano-electrode array sensors and presented the emerging biological applications and their use as portable intelligent devices. The authors addressed the great challenges in electrochemical sensing of trace amounts of molecules and urgent needs in exploring chemical correlations between molecular structure and functions. The minireview aims to promote significantly the understanding of physiological and pathological processes related to matter in chemical movement, offering a unique contribution to chemical and life science research.

There will be a continuous demand for integrated chemical sensor arrays that pair molecular recognition with multimodal transduction. A key index of array-based sensing lies in its performance in practical applications. Challenges still need to be overcome before those laboratory-based optical sensing techniques are widely adapted for field uses (Wang et al., 2017). The development of portable, low-noise, and accurate instrumentation with the capability of onboard and real-time analysis has shown considerable progress over the past decade. It begins to face the increasing challenges for contemporary analytical issues (Jalal et al., 2018). Looking forward, this Research Topic offers an overview of how array-based sensors are constructed and increase the readers’ understanding of ligand-acceptor interactions. We sincerely hope that articles from this special issue could draw much attention from researchers working in relevant fields and promote the studies in the development of new chemical sensing and analytical technology.
